# Treatment of Acanthamoeba neurotrophic corneal ulcer with topical matrix therapy

**DOI:** 10.1186/s12348-015-0048-x

**Published:** 2015-06-21

**Authors:** Antonio Mateo, Beatriz Abadía, Pilar Calvo, Enrique Minguez, Luis Pablo, José Manuel Benitez del Castillo

**Affiliations:** Ophthalmology Department, Miguel Servet University Hospital, Isabel la Catolica 1-3, 50009 Zaragoza, Spain; University of Zaragoza, Zaragoza, Spain; Ophthalmology Department, Lozano Blesa University Clinic Hospital, Zaragoza, Spain; Ophthalmology Department, Clínico San Carlos University Hospital, Madrid, Spain; University Complutense, Madrid, Spain

**Keywords:** Corneal neurotrophic ulcer, Acanthamoeba, RGTA

## Abstract

**Background:**

This study was done to evaluate the visual and anatomical outcomes of topical regenerating agents as a novel therapy for neutrophic corneal ulcer (NCU) secondary to acanthamoeba infection.

**Findings:**

A 20-year-old woman with a history of contact lens wear was referred to our hospital for keratitis after responding poorly to conventional treatment. In vivo confocal microscopy images suggested acanthamoeba keratitis with double-walled cysts in the anterior corneal stroma. Acanthamoeba infection was confirmed by laboratory findings. She was started on 0.1 % propamidine and 0.02 % chlorhexidine drops every hour. The antibiotic and antifungal drops were stopped when bacterial and fungal cultures proved negative. A central neurotrophic corneal ulcers (NCU) appeared, and despite treatment with artificial tears, bandage contact lens, and autologous serum, the ulcer worsened and she was treated with topical CACICOL20 (1 drop every 2 days) for 8 weeks. The corneal defect was completely repaired in 3 weeks. The treatment was well tolerated, and no local or systemic side effects were noted. Visual acuity remained 20/400. Two months later, the defect was still closed and the patient continued with 0.1 % propamidine and 0.02 % chlorhexidine drops, bandage contact lens, artificial tears, and autologous serum.

**Conclusions:**

Topical regenerating agents interact with components of the extracellular matrix, binding matrix proteins and protecting them from proteolysis, restoring the matrix environment, and improving tissue healing. In this case, CALCICOL20 was effective for vision stabilization, wound healing, and was well tolerated for NCU secondary to acanthamoeba infection.

## Findings

Neurotrophic corneal ulcers (NCU) are caused by different ocular and systemic conditions, such as diabetes, herpetic keratitis, corneal dystrophies, and chemical burns. These pathologies have in common a loss of corneal sensitivity leading to impaired healing [1]. The management of NCU is difficult, and one of the most challenging conditions is caused by acanthamoeba infection. This parasite infection is a significant cause of ocular morbidity, but with early and aggressive treatment, acanthamoeba organisms may be eradicated. In some cases, severe inflammation, nerve affectation and necrosis, and substantial loss of corneal tissue may result, requiring more aggressive treatments, such as amniotic membrane transplantation (AMT) or penetrating keratoplasty (PK) [2].

A new approach for the treatment of NCU is topical regenerating agents (RGTA). When artificial tears, autologous serum, or bandage contact lenses fail, RGTA can be used before trying more aggressive treatments. RGTA comprise biopolymers engineered to mimic the structural and functional properties of heparan sulfates. RGTA interact with major components of the extracellular matrix, facilitating corneal healing and protecting against extensive proteolysis caused by the enzymatic activity that characterizes inflammatory, necrotic, or fibrotic sites [3]. We present a case with NCU caused by acanthamoeba infection that was treated successfully with the RGTA, CACICOL20.

**Fig. 1:**
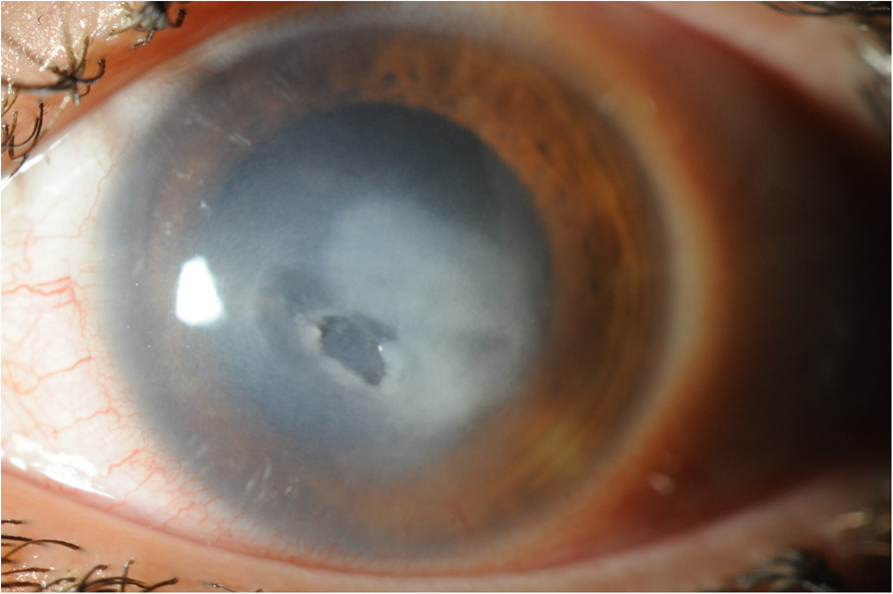
Corneal neurotrophic ulcer secondary to acanthamoeba keratitis

**Fig. 2:**
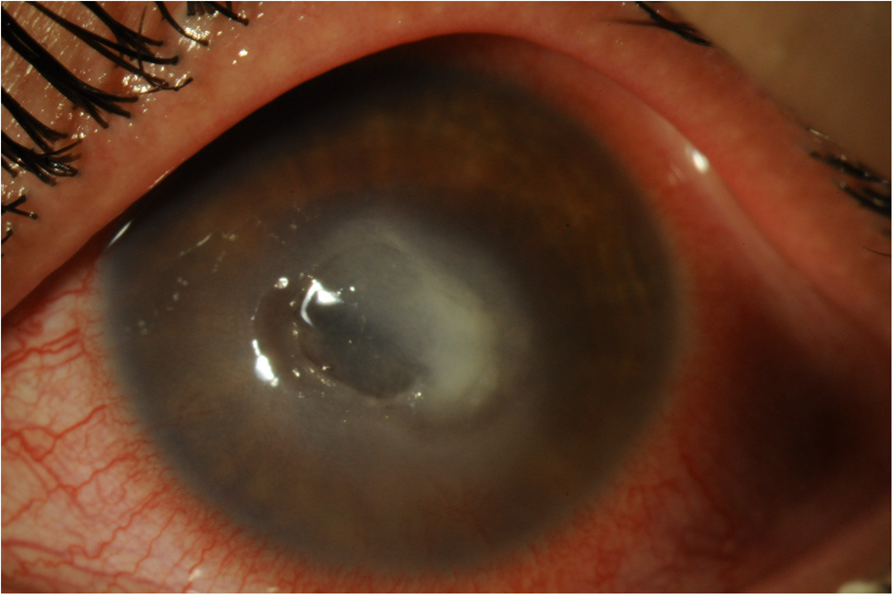
Corneal neurotrophic ulcer worsening despite intensive treatment

**Fig. 3:**
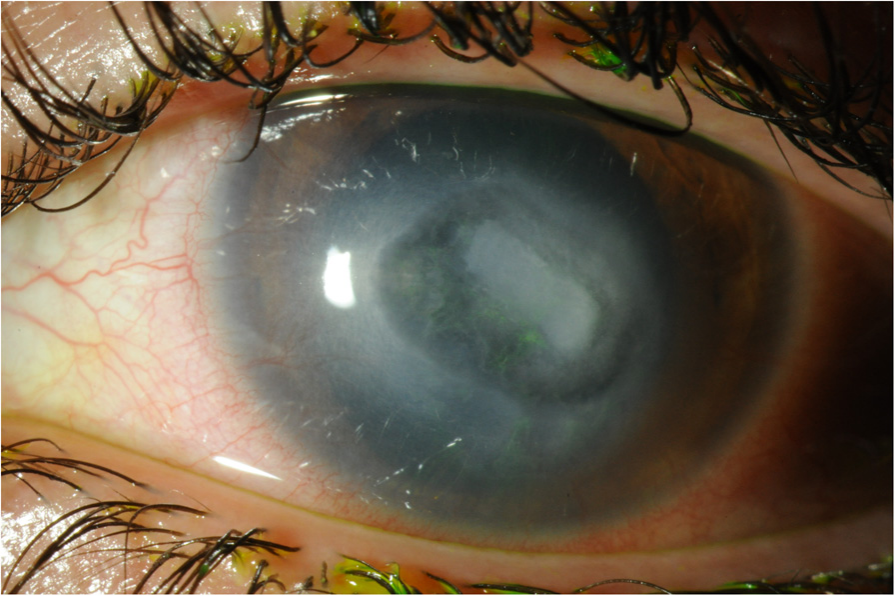
Complete corneal healing after CACICOL20 treatment

A 20-year-old female contact lens wearer was referred to our hospital with a history of keratitis for 2 weeks that responded poorly to conventional treatment with artificial tears and topical erythromycin ointment. The patient complained of cloudy vision and a painful, red left eye. Visual acuity (VA) was 20/20 in the right eye and 20/200 in the left eye. Anterior segment examination with biomicroscopy revealed significant conjunctival injection, epithelial and stromal infiltration, and the anterior chamber was filled with 2+ cells and 1+ flare reaction. Examination of the right eye was unremarkable. Epithelial scraping was obtained and sent to the ocular pathology laboratory. Empirical treatment was started with topical vancomycin, ceftazidime, voriconazole, and amphotericin B and mydriatic agents. Confocal microscopy imaging findings were suggestive for acanthamoeba keratitis with double-walled cysts in the anterior corneal stroma. Six days later, the laboratory confirmed the presence of acanthamoeba. We started treatment with 0.1 % propamidine and 0.02 % chlorhexidine drops every hour. The antibiotic and antifungal drops were stopped when the bacterial and fungal cultures proved negative. Two months later, the cornea was still opacified with infiltration, and a central NCU appeared (Fig. 1). Decreased corneal sensitivity was noted (checked with a cotton swab prior to anesthetic). The surrounding epithelium became loose, Descemet’s membrane developed folds, and the edges of the defect looked smooth and rolled. VA was 20/400. Despite the use of preservative-free artificial tears, a bandage contact lens, and autologous serum, the ulcer worsened (Fig. 2). The patient was then instructed to start using CACICOL20, instilling the eye drops in the morning, as the first eye drop, on alternate days for 8 weeks. After switching therapy, the patient was otherwise treated with preservative-free tears alone. Improvement was observed, and 3 weeks later, the defect was completely repaired (Fig. 3). VA remained 20/400. The treatment was well tolerated, and no local or systemic side effects were noted. Two months later, the defect remained closed and CACICOL20 was stopped. The patient continued with 0.1 % propamidine and 0.02 % chlorhexidine drops, a bandage contact lens, preservative-free artificial tears, and autologous serum.

Trophic ulcerations result from abnormal repair of the corneal epithelium. Management of this type of ulceration is very challenging. Classical treatment includes lubrication with unpreserved artificial teardrops and topical ointment with patching. If this treatment is not effective, autologous serum and topical growth factors can be used. More aggressive treatment includes temporary or permanent tarsorrhaphy, conjunctival flaps, AMT, and lamellar or PK. A new treatment approach for this type of ulcer is matrix RGTA, such as CACICOL20 (RGTA dextran and poly/carboxymethylglucose sulfate). RGTA are biopolymers engineered to replace the components involved in wound healing [3]. CACICOL20 interacts with components of the extracellular matrix, binding matrix proteins, protecting them from proteolysis, restoring the matrix environment, and improving tissue healing.

CACICOL20 is successfully used to treat corneal dystrophies as well as corneal ulcers resistant to standard treatment. Chebbi et al. [4] presented 11 cases with severe corneal dystrophies and painful corneal ulcers with good results in healing. De Monchy et al. [5] reported a case of neurotrophic herpes ulcer successfully treated in 3 weeks with topical RGTA. Kymionis et al. [6] described three cases, including neurotrophic keratopathy, corneal dystrophy, and PK with persistent epithelial defects, that were treated with RGTA, and the epithelial defects were closed in less than 3 weeks. Brignole-Baudouin et al. [7] investigated the effect of RGTA-OTR4120 in an alkali-burned rabbit corneal model and demonstrated that RGTA is well tolerated, and RGTA-treated eyes have fewer signs of inflammation and better reepithelialization. Cejkove et al. [8] also reported a good response in an alkali-burned rabbit corneal model treated with CACICOL20.

To our knowledge, this is the first case of NCU caused by acanthamoeba infection that was successfully treated with CACICOL20. Aifa et al. [9] treated 11 cases of NCU with topical RGTA. They reported that eight cases successfully responded to the treatment, but the other three cases did not show a good response, including a case of acanthamoeba keratitis. This lack of response is contrary to our case.

Hick et al. [10] presented 33 cases of NCU resistant to conventional treatment using AMT; failure was noted in six eyes, one of which had an NCU due to acanthamoeba infection. Acanthamoeba keratitis can be severe and difficult to treat, leading to corneal thinning and risk of perforation, and sometimes requiring PK.

We present a case in which an NCU caused by complications of acanthamoeba infection was successfully treated with CACICOL20. This RGTA could be effective for ulcers resistant to other treatments. This promising drug demonstrates good efficacy and is well tolerated. Further studies are needed, however, to confirm these excellent results.

## Consent

Written informed consent was obtained from the patient for the publication of this report and any accompanying images.
